# Positively Selected Sites at HCMV gB Furin Processing Region and Their Effects in Cleavage Efficiency

**DOI:** 10.3389/fmicb.2017.00934

**Published:** 2017-05-23

**Authors:** Lucas M. Stangherlin, Felipe N. de Paula, Marcelo Y. Icimoto, Leonardo G. P. Ruiz, Maurício L. Nogueira, Antônio S. K. Braz, Luiz Juliano, Maria C. C. da Silva

**Affiliations:** ^1^Center for Natural Sciences and Humanities, Federal University of ABCSanto André, Brazil; ^2^Pasteur InstituteSão Paulo, Brazil; ^3^Department of Biophysics, Paulista Medical School, Federal University of São PauloSão Paulo, Brazil; ^4^Medical School of São José do Rio PretoSão José do Rio Preto, Brazil

**Keywords:** human cytomegalovirus, glycoprotein B, selective pressure, furin cleavage, gB genotypes

## Abstract

Human cytomegalovirus is a ubiquitous infectious agent that affects mainly immunosuppressed, fetuses, and newborns. The virus has several polymorphic regions, in particular in the envelope glycoproteins. The UL55 gene encodes the glycoprotein B that has a variable region, containing a furin cleavage site and according to the variability different genotypes are characterized. Here we investigated variability and existence of selective pressure on the UL55 variable region containing the furin cleavage site in 213 clinical sequences from patients worldwide. We showed the occurrence of positive selective pressure on gB codons 461 and 462, near the furin cleavage site. Cleavage analysis of synthesized peptides demonstrated that most mutations confer better cleavage by furin, suggesting that evolution is acting in order to increase the efficiency cleavage and supporting the hypothesis that gB processing is important in the host. We also demonstrated that peptides containing sequences, that characterize genotypes gB2 and 3, are differentially cleaved by furin. Our data demonstrate for the first time that variability in the cleavage site is related to degree of gB processing by furin.

## Introduction

Human Cytomegalovirus (HCMV) is a ubiquitous human pathogen, present in 40–100% of the global population. In most cases, viral replication is controlled by the host immune system and infection is asymptomatic. HCMV-associated pathogenesis, therefore, predominantly affects immunocompromised individuals such as transplant recipients and AIDS patients (Soderberg-Naucler, 2006; Steininger, 2007), while congenital infection by HCMV is a major cause of birth defects (Gaytant et al., 2002; Cannon, 2009). Additionally, evidence suggests a linkage between HCMV and pathologies such as cancer (Soroceanu and Cobbs, 2011; Dziurzynski et al., 2012; Cobbs et al., 2014) and cardiovascular diseases (Streblow et al., 2001; Yi et al., 2008).

As an opportunistic agent, it is evident that the mechanisms that allow the virus to escape the humoral and cellular immune responses contribute to pathogenesis (Powers et al., 2008; La Rosa and Diamond, 2012; Noriega et al., 2012). Moreover, it is postulated that genetic variability may affect HCMV virulence. HCMV strains have polymorphisms in several regions (Pignatelli et al., 2004; Renzette et al., 2011, 2013; Sijmons et al., 2014) and in particular, the envelope glycoproteins are of interest as they are involved in events such as cell attachment and entry, and are important targets of the host immune system (Isaacson et al., 2008; Vanarsdall and Johnson, 2012).

The effect of genetic variability within the HCMV glycoproteins on many aspects of the viral life cycle has been investigated. One of the most explored polymorphic regions is the UL55 gene that encodes glycoprotein B (gB) (Pignatelli et al., 2004). gB is an essential component of the virus (Isaacson and Compton, 2009), made as a precursor of 160 kDA, that is cleaved by a cellular furin-like protease generating two subunits termed gp58 and gp116. These subunits are present in the viral envelope as a disulfide-linked complex, termed gCI (Britt and Auger, 1986; Spaete et al., 1988; Britt and Vugler, 1989; Vey et al., 1995). gB is the second most abundant protein in the viral particle (Varnum et al., 2004) and during natural infection it is a major target of neutralizing antibodies (Britt et al., 1988; Britt and Vugler, 1990; Marshall et al., 1992; Navarro et al., 1997; Macagno et al., 2010; Potzsch et al., 2011; Spindler et al., 2013). At the viral envelope, it is present as a trimer, similarly to homologs from HSV-1, an alphaherpesvirus, and EBV, a gammaherpesvirus (Sharma et al., 2012). Further, the HCMV gB trimeric domain possesses a high degree of structural homology to the HSV-1 and EBV gB homologs, despite low homology at the sequence level. However, HCMV gB has more glycosylation sites than its other herpesvirus homologs, suggesting that gB uses glycans as shields to protect neutralizing epitopes (Burke and Heldwein, 2015). gB is involved in viral attachment to the cell (Boyle and Compton, 1998), membrane fusion, entry, and cell to cell transfer of the virus (Navarro et al., 1993; Vanarsdall et al., 2008; Isaacson and Compton, 2009). It has also been demonstrated that interaction of gB with PDGFR (Soroceanu et al., 2008), integrins (Feire et al., 2004; Wang et al., 2005), and TLR2 (Boehme et al., 2006) results in diverse intracellular signaling pathways that coordinate cellular events in the host cell.

The gB protein has 906 amino acids, containing a cytoplasmic domain (Cy), a transmembrane region (TM), a membrane proximal region (MP), and a ectodomain (ED) (Sharma et al., 2013). It has three variable regions, which are present in the N-terminus, C-terminus and at the furin cleavage site (aa 460–461) (Chou and Dennison, 1991; Chou, 1992). The gB furin cleavage site is variable, and HCMV strains were first classified into genotypes gB1 through gB4 according to changes in the motif (Chou and Dennison, 1991). More recently, additional rare genotypes were described: gB5, gB6, and gB7 (Trincado et al., 2000).

The clinical relevance of these different genotypes has been evaluated by several studies, in different parts of the world, which investigated a correlation between gB genotypes and tropism, viral loads or clinical disease. While a number of studies found some possible association between gB genotype and infection course (Fries et al., 1994; Shepp et al., 1996; Rasmussen et al., 1997; Meyer-König et al., 1998; Cunha Ade et al., 2002; Wu et al., 2007; Roubalová et al., 2010; Madi et al., 2011; Xia et al., 2012; Vogel et al., 2013), other studies did not found any correlation (Lurain et al., 1999; Trincado et al., 2000; Sarcinella et al., 2002; Carraro and Granato, 2003; Tarragó et al., 2003; Tanaka et al., 2005; Yamamoto et al., 2007; Görzer et al., 2010; Wu et al., 2011; Paradowska et al., 2012). Some reports also indicated that infection with mixed genotypes could be related to disease progression (Sarcinella et al., 2002; Coaquette et al., 2004; Pang et al., 2008; Deckers et al., 2009). However, presently it is not clear if the gB genotype plays a role in disease prognosis. Regarding the requirement for gB cleavage, one study demonstrated that gB processing by furin is not essential for virus production in fibroblasts (Strive et al., 2002). However, the requirement for cleavage *in vivo* and in other cell types was not been determined.

Populations of organisms, including viruses, have genetic variability among their individuals, which can be selected by different environmental pressures. In the context of viral infection, the pressures imposed by the cellular environment positively select the variations that confer a higher fitness to the virus. The selective pressure can be estimated site by site using nucleotide multiple alignment of target regions among different populations and analyzing how it translates to amino acids in the protein primary structure. The identification of genomic regions under positive selection can be measured by estimating the non-synonymous (dN) and synonymous (dS) nucleotide substitutions and their ratio (ω = dN/dS). Rates of (ω) = 1 mean neutral evolution, (ω) < 1 purifying or negative selection, and (ω) > 1 diversifying or positive selection. Biologically, sites under positive selection could indicate regions that might be important for adaptation of the virus, augmenting viral fitness.

In this work, we performed an extensive analysis of the occurrence of positive selection in the variable region of the UL55 ORF that contains the furin cleavage site. We report the occurrence of sites under positive selection pressure. Analysis of the cleavage efficiency by furin, using FRET peptides, indicates that the all mutations on residue 462 (gB2) increase the quality of gB as a furin substrate in terms of processing velocity and afinity while the mutation on residue 461 (gB3) increased the processing velocity, but decreased the affinity. Moreover, sequences that characterize different gB genotypes are differentially cleaved.

## Results

### Determination of the gB Genotype in Clinical Samples and Sequence Data

In order to determine the gB genotypes present in clinical strains obtained from Brazil, and to establish their relationship to sequences from different geographical locations, DNA amplification of the gB variable region was performed in blood samples obtained from 22 renal recipients, 2 allogeneic transplant recipients, 1 premature newborn and tumor tissues tissue from 6 glioblastoma patients (Supplementary Table S1).

The gB2 genotype was the most prevalent (78%), followed by gB3 (18%) and gB1 (4%). Genotypes gB4, gB5, gB6, and gB7 were not found.

### Multiple Alignment and Phylogenetic Analysis

In addition to the sequences obtained from Brazilian patients, 181 sequences from different groups of patients (HIV positive and transplant recipients) from distinct geographic regions were retrieved from GeneBank. The total 213 sequences were aligned and the 100% homologous sequences, at nucleotide level, were excluded (Supplementary Table S1 and **Figure S4**).

**FIGURE 1:**
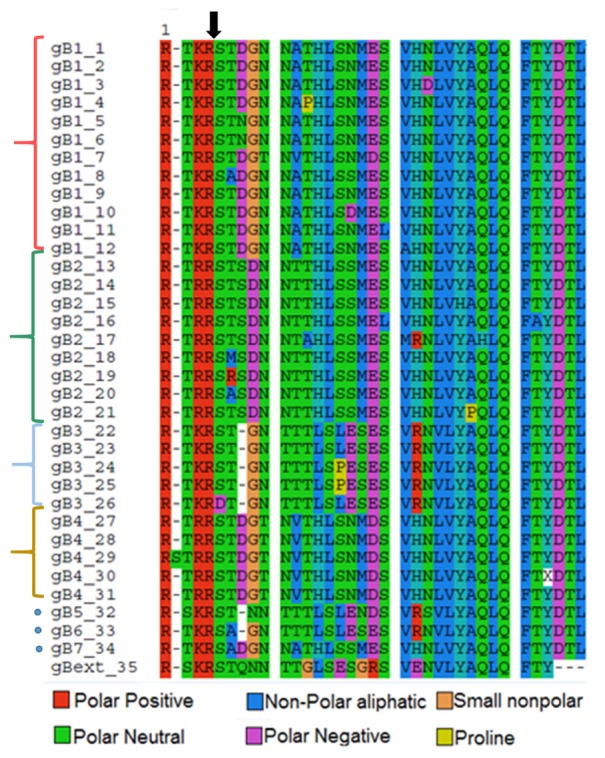
**Multiple sequence alignment of the 35 unique sequences viewed by amino acids.** gB1 to gB4 sequences are grouped (brackets). gB 5, 6, and 7 (dots). gBext_35 corresponds to a Gorilla gorilla cytomegalovirus gB sequence, used as external group to root the phylogenetic tree. Arrow indicates the furin cleavage site. Colors represent amino acids properties as shown in the legend.

**Figure 1** shows the alignment of 36 aa, corresponding to aa 456–492 of gB, in the resulting 35 unique sequences, representing the genotypes 1–4 (brackets) and 5, 6, and 7 (dots).

The cleavage by furin occurs between an arginine (R) and a serine (S) at residues 5 and 6 in the sequence, as indicated by an arrow. As observed the canonical sequence recognized by furin RXR(K)R/S is well conserved, however, mutations outside this region occur frequently and characterize the different genotypes.

**FIGURE 2:**
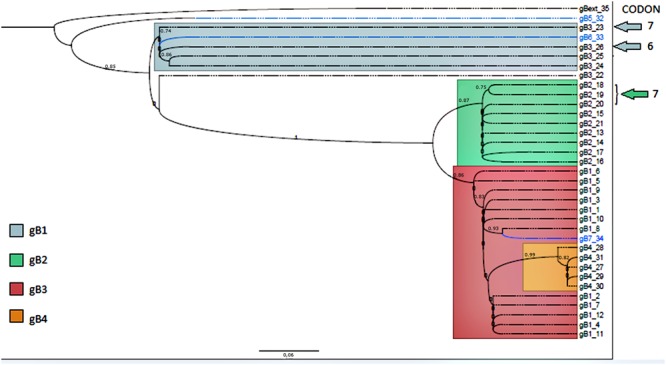
**Phylogenetic tree containing the 35 unique sequences.** Sequences corresponding genotypes gB1 to gB4 are in different colors as indicated. The blue group contains most of the gB3 and gB6; green group contains all the gB2; red group contains all the gB1 and the gB7. Orange group contains all the gB4. The sequences containing positive selective mutations are pointed by arrows and numbered with the codon (in the alignment) which presented the mutation. The bar indicates the phylogenetic distances in terms of genetic change (bar distance = 0.06). Numbers in each node represent genetic change among branches and are expressed as the number of changes divided by the length of the sequence.

To infer a phylogenetic relatedness, among different HCMV isolates worldwide, regarding the variable region of gB gene, a phylogenetic tree was constructed resulting in four distinct groups corresponding to genotypes gB1 to gB4 marked in red, green, dark, blue, and orange, respectively. The genotypes gB5, gB6, and gB7, marked in light blue, do not represent a defined group since there is only one sequence of each variant published. Of note, as previously reported and shown here (**Figure 2**), gB6 and gB7 are close related to gB1 and gB3 (Trincado et al., 2000).

### Detection of Positive Selection

The rate of non-synonymous and synonymous substitutions (dN/dS or B+/α) was used to evaluate the strength and direction of selection. Codons with higher B+/α rates are under a stronger positive selective pressure than codons with lower B+/α rates (Murrell et al., 2012).

The output revealed the occurrence of positive selection on codons 6 and 7 on the alignment (**Figure 1**), which corresponds to codons 461 and 462 of the gB.

Notably, codon 461 is under a significantly stronger positive pressure than codon 462 as demonstrated by its higher B+/α rate (**Table 1**).

**Table 1:** Mixed Effects Model Evolution (MEME) tool output.

*p*-value	α/B+	B+	α	Codon
0.052	24.142	16.782	0.695	6
0.098	6.523	13.913	2.132	7

*Codons with the major selective pressure probability (6 and 7) and their α and *B*+ values.*

**FIGURE 3:**
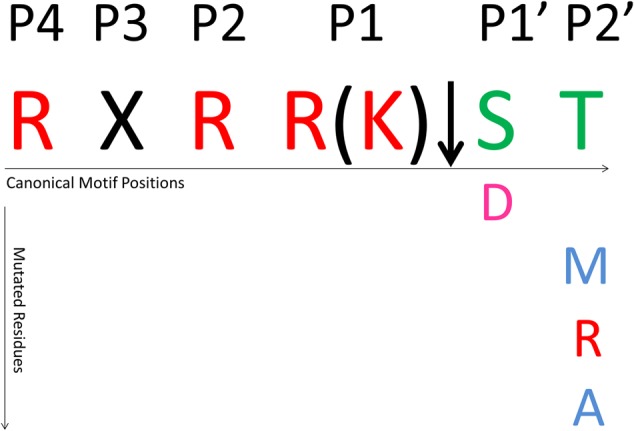
**Furin cleavage site and sites under positive selective pressure.** Red residues are positive polar, pink are negative polar, and green are neutral polar residues.

These two codons are located near the furin cleavage site and correspond to the positions P1′ and P2′, respectively (**Figure 3**), according to the protease-substrate model from Schechter and Berger (1967). For codon 461 only one mutation was observed in genotype 3, where serine (S) is changed to aspartic acid (D), while for codon 462, while threonine (T) was mutated to methionine (M), arginine (R), and alanine (A) in genotype 2. (**Figure 1**). The other regions in the sequence did not exhibit significant positive selection.

**FIGURE 4:**
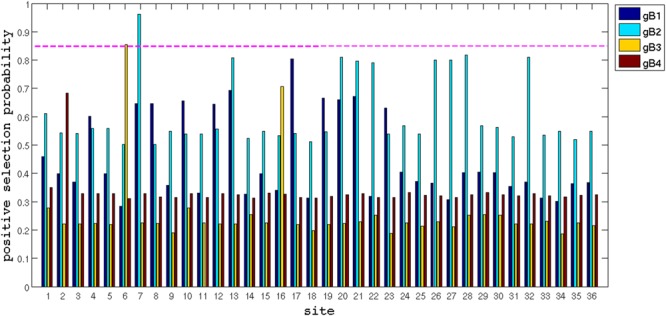
**Bayesian Inference by MrBayes software.** The probability of positive selective pressure (*y*-axis) plot for each site (*x*-axis). The cut-off of probability was 85%.

In order to strength our findings, a second analysis of selective pressure was performed codon by codon for each gB genotype by Bayesian Inference (BI), using the software MrBayes (Ronquist and Huelsenbeck, 2003). The BI confirmed the evidences of positive selection at codons 461 and 462, with a cut-off of 85% of positive selection probability (**Figure 4**).

### Cleavage by Furin

In order to see if the mutations have an influence in cleavage, peptides containing the original reference sequences and their derivative containing positive selected mutations, were synthesized and tested against recombinant human furin. All substrates were cleaved after the double basic residues RR or KR as analyzed by Liquid Chromatography mass spectrometry (LC-MS) (**Supplementary Figures S1**–**S3**). The *k*_cat_/*k*_m_ ratio relates to the catalytic efficiency, meaning how fast in M/s the enzyme reacts with the substrate once it encounters it. As a control, a peptide which contains a mutation previously shown to interfere with furin cleavage (Strive et al., 2002) was tested and as expected no cleavage was observed (**Table 2** and **Supplementary Figure S3**).

**Table 2:** gB2, gB3 peptides, and non-cleaved peptide (NCP) and their respective *k*_cat_, and *km* kinetics parameters.

Peptide	Mutation	*k*_cat_ (1/s)	*SD*	*k*_m_ (μmolar)	*SD*	*k*_cat_/*k*_m_	Sequence name	Genotype
RTRR↓STSDNNT	–	2.24	0.05	3.19	0.06	0.70	gB2_13	2
RTRR↓SRSDNNT	T462R	5.06	0.08	0.23	0.01	22.00	gB2_19	
RTRR↓SMSDNNT	T462M	3.01	0.29	1.65	0.14	1.82	gB2_18	
RTRR↓SASDNNT	T462A	4.06	0.03	0.69	0.04	5.88	gB2_20	
RTKR↓STGNTTT	–	0.25	0.02	0.02	0.01	12.5	gB3_22	3
RTKR↓DTGNTTT	S461D	1.89	0.11	0.43	0.02	4.39	gB3_26	
ATRASTSDNNT	R457A/R460A	–	–	–	–	–	–	NCP

*Standard deviation (SD) were calculated and shown for each parameter.*

As shown in **Table 2** the substitution S461D in position P1′ of genotype 3, results in an increase of *k*_cat_, but a 20 times increase in the *k*_m_, resulting in threefold decrease in *k*_cat_/*k*_m_ ratio. This mutation was found in three renal transplant recipients from São Paulo, Brazil (Supplementary Table S1). Mutations in position P2′, found in genotype 2, are shown in **Table 2**. All substitutions result in increase in *k*_cat_, compared to the reference sequence (gB2_13). The differences in *k*_m_ are notable, in particular in the S462R substitution, which increases 31-fold the *k*_cat_/*k*_m_ ratio. This mutation was found in two allogeneic transplant recipients and eight renal transplant recipients. Mutation S462M was found in one renal transplant recipient and S462A in eight real transplant recipients. All these patients are from São Paulo, Brazil (Supplementary Table S1). Interestingly, all peptides containing the mutations on sites under positive selective pressure are processed at higher velocities (*k*_cat_) in comparison to the reference sequences.

## Discussion

The HCMV genome presents polymorphisms at both intra and inter host levels, and variability is found in many regions, in particular in genes that encode glycoproteins (Renzette et al., 2015). The mutations that arise can be advantageous to the virus resulting in increase in viral fitness and adaptation. In this regard, analysis of variability and selection pressure can identify sites under positive selection that might give benefits to the virus. In fact, HCMV positive selection appears to be a driver of genome evolution, associated with compartmentalization in the host (Renzette et al., 2013). Yet, studies of variability and selective pressure in HCMV sequences from individuals from different geographic regions are scarce.

Here we analyzed the occurrence of positive selection in the gB variable region that contains a furin cleavage site, in sequences from different populations worldwide.

Our analysis shows that the furin cleavage site is highly conserved in patient samples. Nevertheless, positive selection was evident in two codons at positions P1′ (aa 461) and P2′ (aa 462) (**Figure 3**). Notably, mutations that characterize positive selection were not found in regions outside the specific cleavage site.

*In vitro* analysis of the cleavage by furin using FRET peptides indicates that substrates containing the gB2 and gB3 genotypes reference sequences are cleaved with different efficiencies (**Table 2**), and the better-cleaved reference peptide among the genotypes is gB3. Furthermore, three positively selected mutations in genotype 2 significantly increase the efficiency of cleavage, while one mutation in genotype 3 decreases it (**Table 2**). This data reveal that variability in this regions affect cleavage and that mutations under positive selective pressure are indeed modifying the quality of the gB as a furin substrate, either increasing or decreasing the *k*_m_/*k*_cat_ ratio.

Positively selected mutations which alter the gB cleavage kinetics can indicate an augment of the viral fitness. Substrates with an increased *k*_cat_/*k*_m_ ratio are better cleaved by furin, suggesting that gB processing is important for viral replication *in vivo*. We also can speculate that in other context substrates for which the furin have high affinity and low reaction velocity, such as gB3_26, may trap the enzyme, preventing the cleavage of host substrates by furin, which may somehow favor to the virus. In this way, gB could act as a competitive inhibitor of furin. In fact, Bachis et al. (2012) reported that Human Immunodeficience Virus 1 (HIV-1) can reduce the cleavage of host proteins by furin, by reducing cellular levels of the enzyme. It is possible that HCMV acts by a different mechanism by reducing the proteolisis of host proteins.

The role of gB cleavage for HCMV life cycle *in vivo* is not recognized. A study by Strive et al. (2002), indicated that gB cleavage by furin is not essential for *in vitro* replication of the RVHB5 strain in fibroblasts. Analysis in other herpesviruses such as pseudorabies virus (PRV), Epstein-Barr virus (EBV), and BHV-1 gB also concluded that cleavage is not required for virus replication *in vitro* but is important for cell-to-cell fusion (Kopp et al., 1994; Okazaki, 2007; Sorem and Longnecker, 2009).

In murid herpesvirus, gB cleavage is not required for entry in fibroblasts and epithelial cells but is significantly important for entry in macrophages and bone marrow-derived dendritic cells (Glauser et al., 2013). Moreover, cleavage is required for lytic spread of the virus in the lungs (Glauser et al., 2013). Similarly, in varicella-zoster virus (VZV) the furin cleavage motif is not required for viral replication *in vitro* but contributes to pathogenesis in skin tissue *in vivo* (Oliver et al., 2009).

Interestingly, in many viruses that have fusion proteins which are cleaved by furin, such as the influenza hemagglutinin, Newcastle disease virus glycoprotein F0, and human immunodeficiency virus 1 glycoprotein gp160, the cleaved proteins tends to have more hydrophobic residues in P3′–P6′ in comparison to non-viral furin substrates. This fact seems to be related to the fusion efficiency, as the more hydrophobic the residues in P3′–P6′, the more efficient is the fusion to the host cell membrane. On the other hand, high hydrophobicity in this region can reduce the furin cleavage efficiency, since it may restrict access to solvent. Therefore, these viruses have to tune hydrophobicity and furin cleavage efficiency to succeed. One strategy to achieve this fine-tuning is to add small hydrophobic residues such as glycine, alanine, and proline in these positions (Tian, 2009).

In HCMV gB the residues in P3′–P6′ positions are mostly polar, such as aspartic acid (D), asparagine (N), and Threonine (T) (**Figure 1**). In gB1, gB3, gB4, gB6, and gB7, glycine (G), a small non-polar residue, is the only hydrophobic residue present in this region. These observation suggests that in HCMV gB this region is not directly related to host-cell membrane fusion, as its hydrophobicity is low when compared to viruses which have fusion proteins with hydrophobic P3′–P6′ regions. In fact, previous analysis indicate that the HCMV gB fusion loops comprise aa 174, 179, 259, 261, and 262 (Vanarsdall et al., 2016).

Regardless we show that the HCMV gB cleavage site is well conserved implying that cleavage is important for the biological function of gB *in vivo*, similar to other herpesviruses. Perhaps cleavage is required for infection of specific cells types. In fact, the main targets of HCMV *in vivo* are epithelial and endothelial cells and consequently studies need to be done to determine the significance of proteolytic cleavage in these cells.

For the first time we show that variability in the HCMV gB cleavage site is related to distinct degrees of cleavage by furin. All positive selected sites occurred in viral sequences from Brazilian transplanted patients and in more than one host, indicating that the mutations arose and became established in a population. This is the first study showing that selective pressures on the HCMV gB furin cleavage site are driving evolution toward a faster processing of the protein. Our findings open possibilities for further studies, ideally using recombinant viruses containing the mutations, aiming the elucidation of the biological implications of the mutations *in vitro* and *in vivo*.

## Materials and Methods

### Sequence Data Set

A total of 213 sequences corresponding to the gB variable region of HCMV were analyzed in this study. Thirty two sequences were obtained through DNA sequencing of blood from 22 renal transplant, 3 allogeneic transplant recipients, 1 premature newborn and tumor tissues of 6 glioblastoma patients from Brazil. The sequences KX859110 to KX859141, were deposited in NCBI’s GenBank^1^. One hundred eighty one sequences were retrieved from GenBank, which numbered 8 AIDS patients, 8 congenitally infected newborns, 76 renal transplant recipients, 34 hematopoietic cell transplant recipients, 35 unknown cases, and 19 HCMV reference sequences. One sequence from gorilla CMV was used as external group. These sequences represent different geographic regions of the world (France, Brazil, United States, South Korea, and Australia). The accession numbers are shown in Supplementary Table S1.

This study has approval of the research ethics committee (REC) no. 04171312.0.0000.5415 of the Medical School of São José do Rio Preto- FAMERP – São Paulo. The patients were anonymized and the written consent was waived by REC. This study also has approval of the REC 0654/10 of the Neurology Center of Clínicas Hospital – São Paulo. The patients were informed of the procedure and signed a consent form.

### DNA Extraction

DNA from peripheral blood was extracted using the *Wizard^®^ Genomic DNA Purification Kit* (Promega^TM^) and DNA from glioblastoma tumor tissues was extracted using the Pure Link genomic DNA mini kit (Invitrogen), according manufacturer’s instructions.

### Hemi-Nested PCR

To amplify the gB variable region a hemi-nested PCR (nPCR) was performed using primers previously described by Zheng et al. (2002). In the first round of amplification, the reactions were carried out in 50 μL volume containing 2 μL DNA, Taq Polymerase buffer 1x, 2 mM MgCl_2_, 2.5 U Platinum Taq Polymerase High Fidelity (Invitrogen), 200 μM dNTPs, and 0.15 μM of each primer.

The thermo cycling program was set as 94°C for 14 min, 35 cycles at 95°C for 30 s; 56.2°C for 30 s; 72°C for 45 s; 72°C for 10 min. All the reactions were performed at least three times for confirmation and to avoid false positive and negative results. No positive controls were used during the HCMV detection in nPCR to minimize the possibility of laboratory contamination with HCMV DNA. As a positive control for cellular DNA, primers for the Glyceraldehyde 3-phosphate dehydrogenase gene (GAPDH) gene were used (5′-ACC CAC TCC TCC ACC TTT GAC-3′ and 5′-CTG TTG CTG TAG CCA AAT TCG T-3′).

Amplified products were purified using the Illustra GFX PCR DNA and Gel Band Purification Kit (GE Healthcare) and sequenced using the BigDye Terminator v3.1 Cycle Sequencing kit with an ABI-3130 (Applied Biosystems^TM^) at the Serviço de Sequenciamento de DNA–SSDNA IQUSP. Confirmation of the HCMV sequences, and the determination of the gB genotypes were performed using the National Center for Biotechnology Information (NCBI-USA) Blast tool.

### Sequence Alignment and Phylogenetic Analysis

The gB sequences were aligned using the method MUSCLE v3.8.31^2^ (Edgar, 2004) and edited with the Bio edit software^3^ (Hall, 1999). Sequences with 100% similarity were excluded from the analyses.

The best phylogenetic tree substitution method was determined by performing tests on the jModelTest2 software^4^ (Darriba et al., 2012). A phylogenetic tree of the 35 resulting unique sequences was constructed using the GTR model of the software PhyML^5^ (Guindon et al., 2010).

The gB genotypes were assigned by comparison of obtained sequences with reference sequences previously published (Supplementary Table S1).

### Positive Selection Analysis

The search for positive selection was performed using the Mixed Effects Model Evolution (MEME) (Murrell et al., 2012) via Datamonkey web Server (Pond and Frost, 2005)^6^, which detect episodic diversifying selection affecting individual codon sites in branch by branch. Selective pressure is estimated codon-by-codon based in the ratio of mutations that lead to non-synonymous (dN) or synonymous (dS) substitutions. The algorithm attributes different weights for each substitution, according to the probability of occurrence and their impact in the protein translation. In this manner scores are calculated for synonymous (α) and non-synonymous (β+) substitutions and the ratio is used to estimate the selective pressure site by site. A second analysis of the probability of selection was done for each gB genotype by BI, using the software MrBayes (Ronquist and Huelsenbeck, 2003) with a 85% probability cut-off (**Figure 4**).

### Recombinant Furin Production

Recombinant human furin was expressed and purified as previously described (Kacprzak et al., 2004). The enzyme concentration was determined by active site titration using the inhibitor decanoyl-RVKR-CMK inhibitor in 10 mM MES buffer, 1 mM CaCl2, pH 7.0 and the substrate Abz-GIRRKRSVSHQ-EDDnp.

### FRET-Peptide Synthesis and Enzymatic Kinetics Assays

Seven synthetic peptides were obtained by solid-phase strategy, as previously described (Izidoro et al., 2010) using the Fmoc-procedure in an automated multiple solid-phase peptide synthesizer (PSSM 8 system, Shimadzu, Japan) (Hirata et al., 1995; Korkmaz et al., 2008). Peptides were prepared in dimethylformamide and stock concentrations measured spectrophotometrically at 365 nm (*e* = 17,300 M^-1^.cm^-1^). The quantum yields of the FRET peptides and the products of their hydrolysis were unchanged in all tested conditions.

The FRET peptides were assayed in 10 mM MES, 1 mM CaCl2, pH 7.0, using a F-2500 spectrofluorimeter (Hitachi, Tokyo, Japan), at 37°C (Izidoro et al., 2010), as previously described. Fluorescence changes were monitored continuously at λex = 320 nm and λem = 420 nm. The enzyme concentrations for initial rate determinations were chosen at a level intended to hydrolyze less than 5% of the amount of added substrate over the time course of data collection.

Three kinetic parameters from the Michaelis-Menten model were analyzed, representing enzyme-substrate (*k*_m_), the catalysis constant (*k*_cat_) and the *k*_cat_/*k*_m_ ratio. The kinetic parameters *k*_m_ and *k*_cat_ were calculated by non-linear regression using Grafit software (Erithacus Software, Horley, Surrey, UK). *k*_m_, *k*_cat_, and standard errors (less than 10%) were calculated using Grafit software (v.5.0.13, Erithacus Software, Horley, Surrey, UK) with a non-linear least squares Michaelis-Menten fit associated with a matrix invertion fitting routine to refine *k*_cat_, *k*_m_ and allowing estimation of the errors. Standard deviations greater than 10% are due to low values obtained.

## Author Contributions

All the authors contributed with: substantial contributions to the conception or design of the work; or the acquisition, analysis, or interpretation of data for the work; drafting the work or revising it critically for important intellectual content; final approval of the version to be published; agreement to be accountable for all aspects of the work in ensuring that questions related to the accuracy or integrity of any part of the work are appropriately investigated and resolved.

## Conflict of Interest Statement

The authors declare that the research was conducted in the absence of any commercial or financial relationships that could be construed as a potential conflict of interest.

## Footnotes

1 http://www.ncbi.nlm.nih.gov

2 http://www.ebi.ac.uk/Tools/msa/muscle

3 http://www.mbio.ncsu.edu/BioEdit/bioedit.html

4 https://code.google.com/p/jmodeltest2/

5 http://www.atgc-montpellier.fr/phyml/

6 http://www.datamonkey.org

## Supplementary Material

FIGURE S1 **HPLC of furin-processed FRET substrates. Each substrate was incubated with/without furin for 12 h and further analyzed by HLPC.** Substrate and products were followed by fluorescence (Abz, ex_320 nm_; em_420 nm_) quenching (Eddnp, uv = 365 nm) indicating single cleavage site for all tested sequences. **(A)** gB3_22. **(B)** gB_20. **(C)** gB3_26 **(D)** gB2_18. **(E)** gB2_13. **(F)** gB2_19.

FIGURE S2 **Mass spectra of furin-processed substrates.** Furin (50 nM) were incubated with each substrate for 12 h and further analyzed by Maldi-TOF (Bruker Daltonics, Germany). **(A)** gB3_22 substrate and products (1549.3, 678.7, and 888.7 Da). **(B)** gB2_13 (1634.4, 706.7, and 945.7 Da). **(C)** gB_20 (1604.3, 706.7, and 915.6 Da). **(D)** gB2_18 (1664.4, 706.7, and 975.8 Da) **(E)** gB3_26 (1577.3, 678.7, and 916.7 Da). **(F)** gB2_19 (1689.4, 706.7, and 1000.8 Da).

FIGURE S3 **Maldi-TOF spectrum of the synthetic FRET peptide ATRASTSDNNT (Strive et al., 2002), not processed by furin.** FRET ATRASTSDNNT (5 μM, 1,592.2 Da) was tested as a furin substrate in spectrofluorimeter and Maldi-TOF after 2 h of incubation with furin.

FIGURE S4 **Multiple sequence alignment of the 35 unique sequences viewed by nucleotides**.
