# Supplementary figures and images for: Positively Selected Sites at HCMV gB Furin Processing Region and Their Effects in Cleavage Efficiency
Source: Front Microbiol. 2017 May 23;8:934. doi: 10.3389/fmicb.2017.00934 (PMC5441137; doi:10.3389/fmicb.2017.00934)

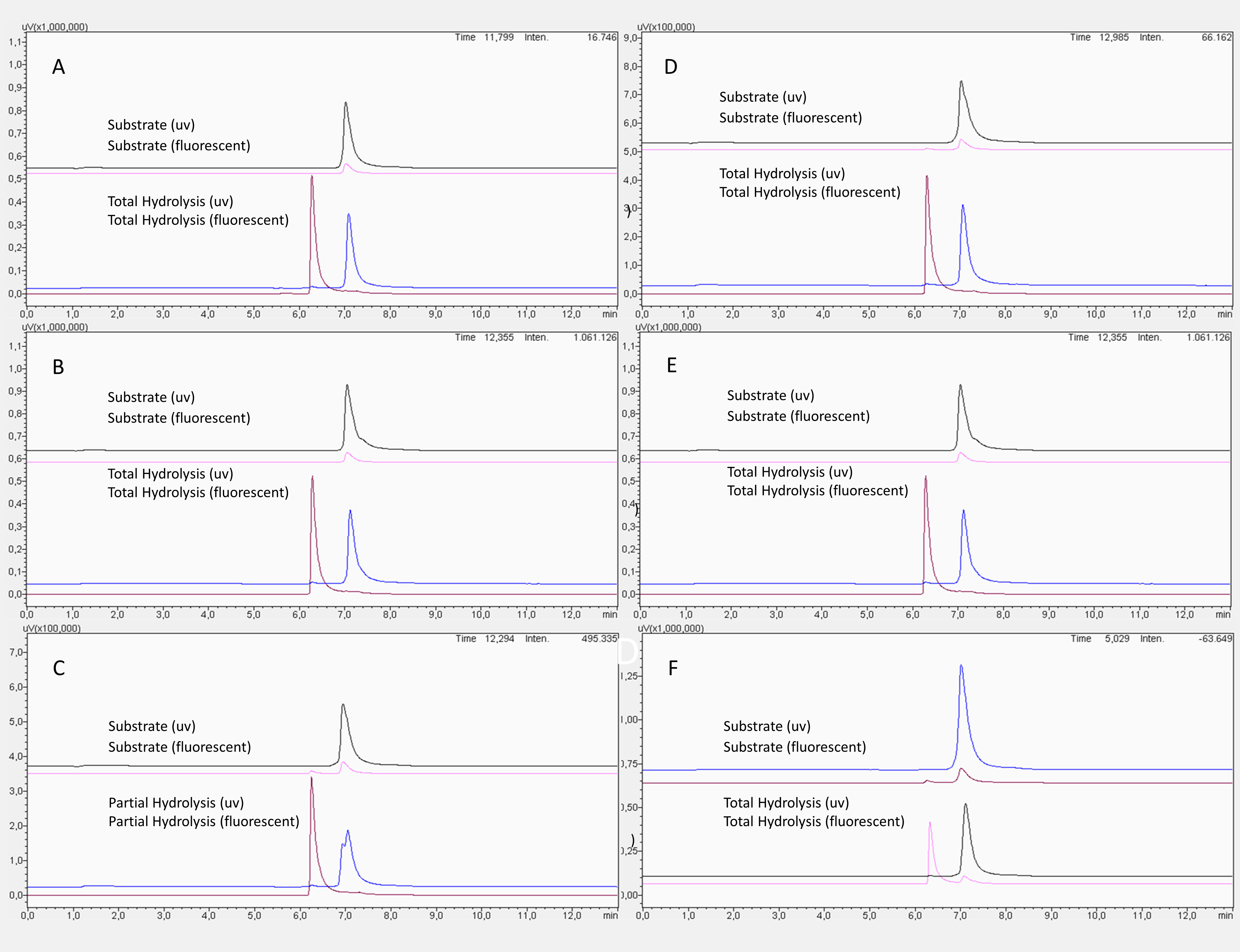

Supplement: FIGURE S1 — HPLC of furin-processed FRET substrates. Each substrate was incubated with/without furin for 12 h and further analyzed by HLPC. Substrate and products were followed by fluorescence (Abz, ex320 nm; em420 nm) quenching (Eddnp, uv = 365 nm) indicating single cleavage site for all tested sequences. (A) gB3_22. (B) gB_20. (C) gB3_26 (D) gB2_18. (E) gB2_13. (F) gB2_19. [file Image_1.TIF]

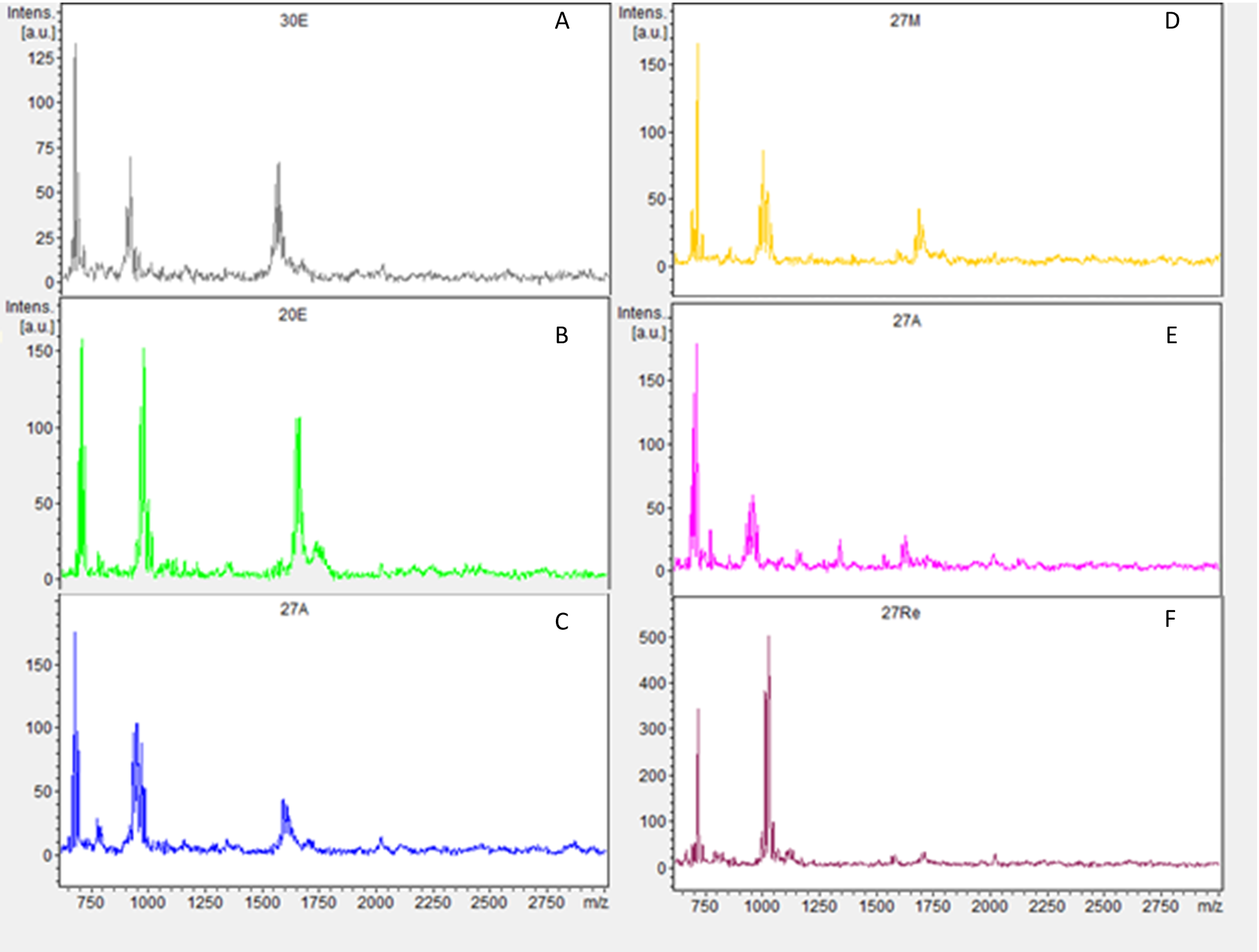

Supplement: FIGURE S2 — Mass spectra of furin-processed substrates. Furin (50 nM) were incubated with each substrate for 12 h and further analyzed by Maldi-TOF (Bruker Daltonics, Germany). (A) gB3_22 substrate and products (1549.3, 678.7, and 888.7 Da). (B) gB2_13 (1634.4, 706.7, and 945.7 Da). (C) gB_20 (1604.3, 706.7, and 915.6 Da). (D) gB2_18 (1664.4, 706.7, and 975.8 Da) (E) gB3_26 (1577.3, 678.7, and 916.7 Da). (F) gB2_19 (1689.4, 706.7, and 1000.8 Da). [file Image_2.TIF]

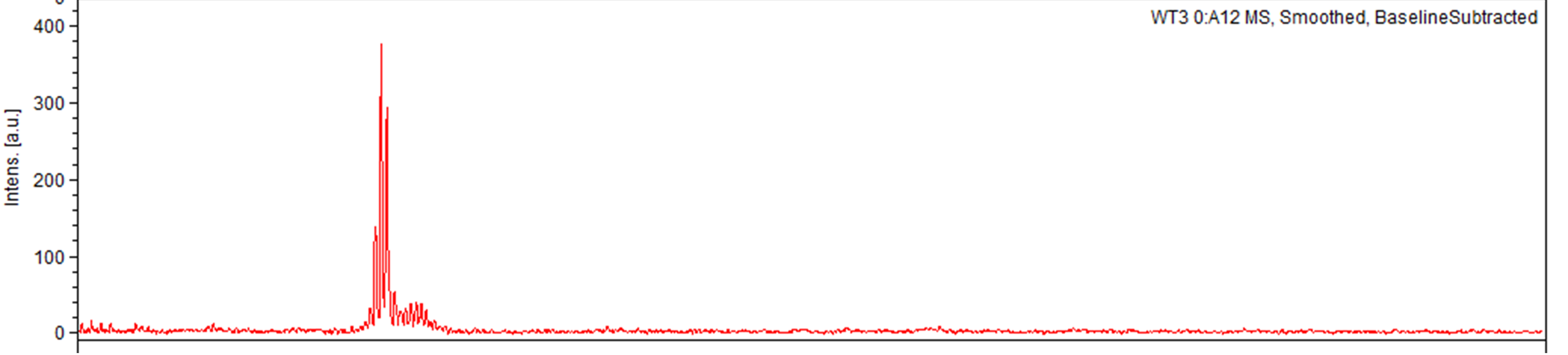

Supplement: FIGURE S3 — Maldi-TOF spectrum of the synthetic FRET peptide ATRASTSDNNT (Strive et al., 2002), not processed by furin. FRET ATRASTSDNNT (5 μM, 1,592.2 Da) was tested as a furin substrate in spectrofluorimeter and Maldi-TOF after 2 h of incubation with furin. [file Image_3.TIF]

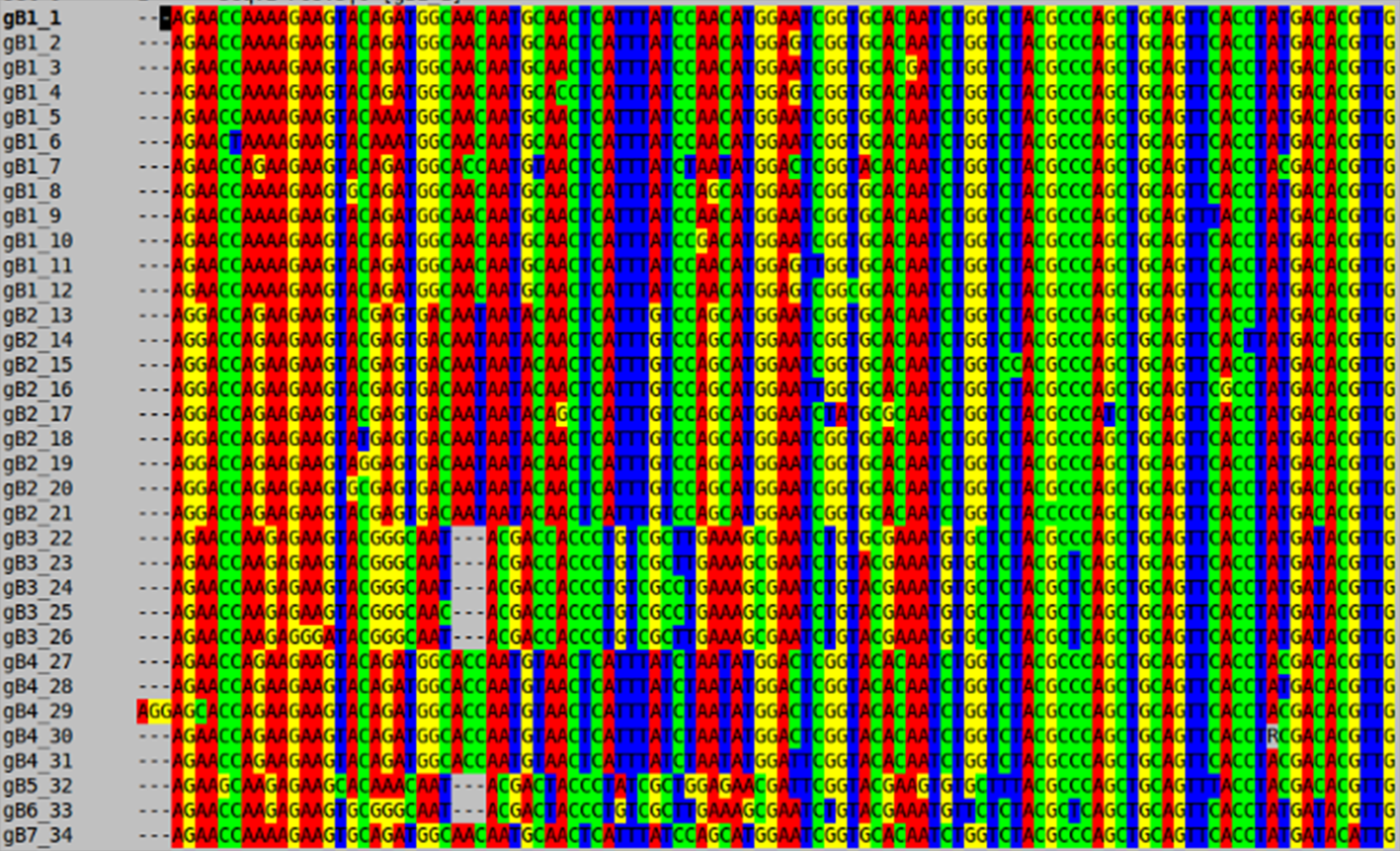

Supplement: FIGURE S4 — Multiple sequence alignment of the 35 unique sequences viewed by nucleotides. [file Image_4.TIF]
