# Supplementary material for: Positively Selected Sites at HCMV gB Furin Processing Region and Their Effects in Cleavage Efficiency
Source: Front Microbiol. 2017 May 23;8:934. doi: 10.3389/fmicb.2017.00934 (PMC5441137; doi:10.3389/fmicb.2017.00934)
Supplement: Supplementary file 5 [file Table_1.PDF]

Table S1. The 35 gB unique sequences groups. These groups correspond to the sequences on the multiple amino acid alignment and comprehends all the 212 sequences. All groups' components are shown with their respective GenBank accession number.

Group	Name	Genotype	*Samples number*	Components
1	gB1_1	1	*14*	France_x_1_9 (FR677233.1)<br>France_x_1_8(FR677240.1)<br>France_x_1_10(FR677227.1)<br>France_x_1_11(FR677226.1)<br>France_x_1_12(FR677224.1)<br>France_x_1_13(FR677223.1)<br>France_x_1_14(FR677221.1)<br>France_x_1_15(FR677220.1)<br>France_Congenital_1_16 (FJ790956.1)<br>France_Congenital_1_17 (FJ790954.1)<br>France_Congenital_1_18 (FJ790953.1)<br>France_Congenital_1_19 (FJ790952.1)<br>France_Congenital_1_20(FJ790951.1)<br>France_Congenital_1_21 (FJ790950.1)
2	gB1_2	1	*15*	France_x_1_22 (FR677242.1)<br>France_x_1_23 (FR677231.1)<br>France_Congenital_1_24 (FJ790961.1)<br>SaoPaulo_RenalTransp_1_31 (AY186165.1)<br>SaoPaulo_RenalTransp_1_32 (AY186163.1)<br>SaoPaulo_RenalTransp_1_33 (AY186160.1)<br>SaoPaulo_RenalTransp_1_34 (AY186142.1)<br>SaoPaulo_RenalTransp_1_35 (AY186139.1)<br>SaoPaulo_RenalTransp_1_36 (AY186100.1)<br>SaoPaulo_RenalTransp_1_37 (AY186098.1)<br>SaoPaulo_RenalTransp_1_38 (AY186094.1)<br>SaoPaulo_RenalTransp_1_39 (AY186134.1)<br>SaoPaulo_RenalTransp_1_40 (AY186167.1)<br>SaoPaulo_RenalTransp_1_41 (AY186151.1)<br>SaoPaulo_RenalTransp_1_42 (AY186144.1)
3	gB1_3	1	*17*	SaoPaulo_RenalTransp_1_44 (AY186101.1)<br>SaoPaulo_RenalTransp_1_45 (AY186135.1)<br>SaoPaulo_RenalTransp_1_46 (AY186125.1)<br>SaoPaulo_RenalTransp_1_47 (AY186186.1)<br>SaoPaulo_RenalTransp_1_48 (AY186185.1)<br>SaoPaulo_RenalTransp_1_49 (AY186183.1)<br>SaoPaulo_RenalTransp_1_50 (AY186176.1)<br>SaoPaulo_RenalTransp_1_51 (AY186175.1)<br>SaoPaulo_RenalTransp_1_52 (AY186157.1)<br>SaoPaulo_RenalTransp_1_53 (AY186156.1)<br>SaoPaulo_RenalTransp_1_54 (AY186137.1)<br>SaoPaulo_RenalTransp_1_55 (AY186115.1)<br>SaoPaulo_RenalTransp_1_56 (AY186104.1)<br>SaoPaulo_RenalTransp_1_57 (AY186166.1)<br>SaoPaulo_RenalTransp_1_60 (AY186116.1)<br>SaoPaulo_RenalTransp_1_61 (AY186184.1)<br>SaoPaulo_RenalTransp_1_62 (AY186152.1)

4	gB1_4	1	*1*	SaoPaulo_RenalTransp_1_64 (AY186173.1)
5	gB1_5	1	*7*	SaoPaulo_RenalTransp_1_66 (AY186095.1)<br>SaoPaulo_RenalTransp_1_67 (AY186132.1)<br>SaoPaulo_RenalTransp_1_68 (AY186123.1)<br>SaoPaulo_RenalTransp_1_69 (AY186188.1)<br>SaoPaulo_RenalTransp_1_71 (AY186149.1)<br>SaoPaulo_RenalTransp_1_72 (AY186118.1)<br>SaoPaulo_RenalTransp_1_73 (AY186180.1)
6	gB1_6	1	*7*	SaoPaulo_RenalTransp_1_74 (AY186177.1)<br>SaoPaulo_RenalTransp_1_75 (AY186162.1)<br>SaoPaulo_RenalTransp_1_76 (AY186154.1)<br>SaoPaulo_RenalTransp_1_77 (AY186141.1)<br>SaoPaulo_RenalTransp_1_79 (AY186136.1)<br>SaoPaulo_RenalTransp_1_80 (AY186109.1)<br>SaoPaulo_RenalTransp_1_81 (AY186117.1)
7	gB1_7	1	*4*	SaoPaulo_RenalTransp_1_83 (AY186130.1)<br>USA_RecSoro_1_3(GU365817.1)<br>SouthKorea_HSCTblf_1_7 (FJ820991.1)<br>SouthKorea_HSCTblood_1_8(FJ820979.1)
8*	gB1_8	1	*1*	SouthKorea_HSCTblood_1_9 (FJ820964.1)
9	gB1_9	1	*3*	SouthKorea_HSCTblood_1_10 (FJ820989.1)<br>SouthKorea_HSCTblf_1_11 (FJ820987.1)<br>SouthKorea_HSCTblf_1_12 (FJ820984.1)
10	gB1_10	1	*16*	SouthKorea_HSCTblood_1_13 (FJ820978.1)<br>SouthKorea_HSCTblood_1_14 (FJ820975.1)<br>SouthKorea_HSCTblood_1_15 (FJ820969.1)<br>SouthKorea_HSCTblood_1_16 (FJ820966.1)<br>SouthKorea_HSCTblood_1_17 (FJ485728.1)<br>SouthKorea_HSCTblood_1_18 (GQ166965.1)<br>SouthKorea_HSCTblood_1_20 (FJ820972.1)<br>SouthKorea_HSCTblood_1_21 (FJ820971.1)<br>SouthKorea_HSCTblood_1_22 (FJ820968.1)<br>SouthKorea_HSCTblood_1_23 (FJ820963.1)<br>SouthKorea_HSCTblood_1_24 (FJ820961.1)<br>SouthKorea_HSCTblood_1_26 (GQ166969.1)<br>SouthKorea_HSCTblood_1_27 (GQ166967.1)<br>SouthKorea_HSCTblood_1_28 (FJ820983.1)<br>SouthKorea_HSCTblood_1_29 (FJ820982.1)<br>SouthKorea_HSCTblood_1_30 (FJ820980.1)
11	gB1_11	1	*1*	SouthKorea_HSCTblood_1_31 (FJ820976.1)
12	gB1_12	1	*19*	SouthKorea_HSCTblood_1_32 (FJ820974.1)<br>SouthKorea_HSCTblood_1_33 (FJ820970.1)<br>SouthKorea_HSCTblood_1_34 (FJ820967.1)<br>SouthKorea_HSCTblood_1_35 (FJ820962.1)<br>SouthKorea_HSCTblood_1_36 (GQ166966.1)<br>C_Australia_x_1_5 (AF210782.1)<br>C_Australia_x_1_6 (AF210776.1)<br>C_Australia_x_1_7 (AF210775.1)<br>C_Australia_x_1_8 (AF210774.1)<br>Ref_x_1_18<br>Ref_3301_1_20<br>Target_TranspRenal_1_18<br>Ref_towne_1_2<br>Ref_AF1_1_6

				Ref_Merlin_1_7<br>Ref_U8_1_8<br>Ref_3157_1_9<br>Ref_x_1_10<br>Target_TranspRenal_1_21
13	gB2_13	2	*7*	USA_RecSoro_2_1 (GU365821.1)<br>USA_RecSoro_2_2(GU365820.1)<br>France_x_2_1 (FR677241.1)<br>France_x_2_2 (FR677239.1)<br>France_x_2_3 (FR677237.1)<br>France_x_2_4 (FR677234.1)<br>France_x_2_5 (FR677222.1)
14	gB2_14	2	*32*	France_x_2_7 (FR677225.1)<br>SaoPaulo_RenalTransp_2_1 (AY186148.1)<br>SaoPaulo_RenalTransp_2_2 (AY186122.1)<br>SaoPaulo_RenalTransp_2_3 (AY186114.1)<br>SaoPaulo_RenalTransp_2_4 (AY186102.1)<br>SaoPaulo_RenalTransp_2_5 (AY186099.1)<br>SaoPaulo_RenalTransp_2_6 (AY186097.1)<br>SaoPaulo_RenalTransp_2_7 (AY186178.1)<br>SaoPaulo_RenalTransp_2_8 (AY186107.1)<br>SaoPaulo_RenalTransp_2_9 (AY186189.1)<br>SaoPaulo_RenalTransp_2_10 (AY186172.1)<br>SaoPaulo_RenalTransp_2_11 (AY186168.1)<br>SaoPaulo_RenalTransp_2_12 (AY186164.1)<br>SaoPaulo_RenalTransp_2_13 (AY186181.1)<br>SaoPaulo_RenalTransp_2_14 (AY186159.1)<br>SaoPaulo_RenalTransp_2_15 (AY186153.1)<br>SaoPaulo_RenalTransp_2_16 (AY186161.1)<br>SaoPaulo_RenalTransp_2_17 (AY186131.1)<br>SaoPaulo_RenalTransp_2_18 (AY186103.1)<br>SaoPaulo_RenalTransp_2_19 (AY186150.1)<br>SaoPaulo_RenalTransp_2_20 (AY186187.1)<br>SaoPaulo_RenalTransp_2_21 (AY186126.1)<br>SaoPaulo_RenalTransp_2_22 (AY186105.1)<br>SaoPaulo_RenalTransp_2_23 (AY186110.1)<br>SaoPaulo_RenalTransp_2_24 (AY186174.1)<br>SaoPaulo_RenalTransp_2_25 (AY186169.1)<br>SaoPaulo_RenalTransp_2_26 (AY186124.1)<br>SaoPaulo_RenalTransp_2_27 (AY186120.1)<br>SaoPaulo_RenalTransp_2_28 (AY186113.1)<br>SaoPaulo_RenalTransp_2_29 (AY186179.1)<br>SouthKorea_HSCTblood_2_1 (FJ820981.1)<br>SouthKorea_HSCTblf_2_2(FJ820993.1)
15	gB2_15	2	*2*	SouthKorea_HSCTblf_2_3(FJ820992.1)<br>SouthKorea_HSCTblood_2_4(FJ820977.1)
16	gB2_16	2	*1*	SouthKorea_HSCTblood_2_5(GQ166964.1)
17	gB2_17	2	*8*	SouthKorea_HSCTblood_2_6(FJ820965.1)<br>C_Australia_x_2_1 (AF210781.1)<br>C_Australia_x_2_2 (AF210780.1)

				C_Australia_x_2_3 (AF210779.1)<br>C_Australia_x_2_4 (AF210777.1)<br>Ref_x_2_1<br>Ref_x_2_4<br>Target_RnPrematuro_2_1
18*	gB2_18	2	*1*	Target_TranspRenal_2_2
19*	gB2_19	2	*10*	Target_Alogenico_2_3<br>Target_TranspRenal_2_4<br>Target_TranspRenal_2_5<br>Target_TranspRenal_2_6<br>Target_TranspRenal_2_7<br>Target_TranspRenal_2_8<br>Target_TranspRenal_2_9<br>Target_Alogenico_2_10<br>Target_TranspRenal_2_11<br>Target_TranspRenal_2_12
20*	gB2_20	2	*8*	Target_TranspRenal_2_13<br>Target_TranspRenal_2_14<br>Target_TranspRenal_2_15<br>Target_TranspRenal_2_16<br>Target_TranspRenal_2_17<br>Target_TranspRenal_2_20<br>Target_TranspRenal_2_24<br>Target_TranspRenal_2_26
21	gB2_21	2	*7*	Target_Tumor_2_1<br>Target_Tumor_2_2<br>Target_Tumor_2_3<br>Target_Tumor_2_4<br>Target_Tumor_2_5<br>Target_Tumor_2_6<br>Ref_JP_2_19
22	gB3_22	3	*1*	C_Australia_x_3_14 (AF210778.1)
23	gB3_23	3	*3*	C_Australia_x_3_15 (AF210785.1)<br>C_Australia_x_3_16 (AF210784.1)<br>C_Australia_x_3_17 (AF210783.1)
24	gB3_24	3	*1*	Ref_x_3_3
25	gB3_25	3	*6*	Ref_toledo_3_5<br>Ref_HAN38_3_11<br>Ref_JHC_3_12<br>Ref_HAN13_3_15<br>Ref_HAN20_3_16<br>Ref_x_3_17
26*	gB3_26	3	*3*	Target_TranspRenal_3_22<br>Target_TranspRenal_3_23<br>Target_TranspRenal_3_25
27	gB4_27	4	*6*	France_x_4_24 (FR677238.1)<br>France_x_4_25(FR677235.1)<br>France_x_4_26(FR677232.1)<br>France_x_4_27(FR677229.1)<br>France_x_4_28(FR677228.1)

				France_Congenital_4_29(FJ790960.1)
28	gB4_28	4	*3*	USA_AIDS_4_1(U88700.1)<br>USA_AIDS_4_2(U88704.1)<br>USA_AIDS_4_3(U88703.1)
29	gB4_29	4	*1*	USA_AIDS_4_4(U88702.1)
30	gB4_30	4	*1*	USA_AIDS_4_5(U88701.1)
31	gB4_31	4	*2*	SaoPaulo_RenalTransp_4_86(AY186112.1)<br>SaoPaulo_RenalTransp_4_87(AY186111.1)
32	gB5_32	5	*1*	Ref_x_5_13
33	gB6_33	6	*1*	C_Australia_x_6_18 (AF210790.1)
34	gB7_34	7	*1*	C_Australia_x_7_9 (AF210791.1)
35	gBext_35	Ext	*1*	Ext_GMV_x_1
